# Transcriptional Response of Rice Mesocotyl Elongation to Sowing Depth and Identification of Key Regulatory Factors

**DOI:** 10.3390/genes17040382

**Published:** 2026-03-27

**Authors:** Ya Wang, Dong Liu, Mengjuan Ma, Ming Li, Jing Fu, Fengjiang Yu, Qiulin Li, Yuetao Wang, Fuhua Wang, Liyu Huang, Haiqing Yin

**Affiliations:** 1 Cereal Crops Research Institute, Henan Academy of Agricultural Sciences, Zhengzhou 450002, China; wangya840212@163.com (Y.W.); liudongzh@hnagri.org.cn (D.L.);; 2 School of Agriculture, Yunnan University, Kunming 650091, China

**Keywords:** rice, mesocotyl, transcriptome analysis, key gene

## Abstract

Background/Objectives: Having longer mesocotyls is beneficial for the deep-sowing tolerance of rice, which is important for seedling establishment. Methods: Here, we performed transcriptome analysis of the elongating mesocotyl of Zhengdao 209 in response to three different sowing depths to identify the pivotal genes regulating rice mesocotyl elongation. Results: Three groups with different mesocotyl lengths were compared using transcriptome analysis, and 60 common differentially expressed genes were detected. Gene Ontology and Kyoto Encyclopedia of Genes and Genomes enrichment analyses revealed that these genes are primarily involved in phenylpropanoid biosynthesis, cutin suberine and wax biosynthesis, the plant mitogen-activated protein kinase signaling pathway, diterpenoid biosynthesis, cyanoamino acid metabolism, carbon fixation in photosynthetic organisms, flavonoid biosynthesis, and glutathione metabolism. Furthermore, weighted gene co-expression network and hierarchical clustering analyses showed that most of the differentially expressed genes are implicated in phenylpropanoid biosynthesis, carbon metabolism, photosynthesis antenna proteins, and plant–pathogen interactions. Among the genes involved in phenylpropanoid biosynthesis processes, the expression levels of *OsPHT3* and *LOC_Os04g59260* increased, while *OsCCR1*, *OsPGIP4*, and *LOC_Os01g45110* expression decreased with increasing sowing depth. Among the genes involved in the mitogen-activated protein kinase signaling pathway, the expression levels of *LOC_Os07g03319* and *LOC_Os07g03580* increased, while *LOC_Os07g03409* decreased with increasing sowing depth. Among the genes involved in diterpenoid biosynthesis processes, the expression levels of *OsCYP76M5* and *OsCYP71Z2* decreased, while *OsCYP71Z21* increased with increasing sowing depth. Furthermore, the expression levels of these genes were analyzed using quantitative real-time polymerase chain reaction, which confirmed the transcriptome analysis results. Conclusions: This study identified candidate genes governing rice mesocotyl length and provides novel insights into the molecular regulatory mechanisms underlying mesocotyl elongation in rice.

## 1. Introduction

Rice (*Oryza sativa* L.) is one of the most important food crops in the world, supporting approximately 56% of the global population [1]. Therefore, stabilizing rice-planting areas and grain yield is crucial for national food security in China. Rice cultivation mainly consists of two methods: seedling transplantation and direct seeding. With environmental changes and technological advancements, direct seeding has been expanded in an increasing number of regions due to its lower water consumption and labor input [1,2]. However, in practical production, direct rice seeding, particularly dry direct seeding, faces a range of challenges, including low emergence rates, uneven seedling emergence, and poor growth vigor with most modern rice varieties under deep-sowing conditions caused by agronomic problems such as non-leveled land [3]. Adequate seedling establishment is critical for high and stable yields of direct-seeded rice; thus, delayed emergence and poor seedling establishment are major restricting factors in the large-scale promotion and application of direct-seeded rice [4].

The rice mesocotyl is defined as the embryonic tissue located between the coleoptilar node and the basal part of the seminal root in young seedlings [5]. Mesocotyl elongation is the primary dynamic contributing to pushing the shoot tip above the soil surface during germination, and a significant positive correlation has been demonstrated between rice soil-penetrating emergence (deep-sowing tolerance) capacity and mesocotyl length (ML) [3]. Varieties with long mesocotyl can be used to improve rice seedling emergence rates under deep sowing conditions. Thus, ML has been proposed as a pivotal trait for dry direct-seeding rice production.

Mesocotyl elongation in rice is a complex trait regulated by genetic factors and environmental conditions. Regarding environmental factors, mesocotyl elongation is influenced by light, temperature, soil conditions, and plant hormones [6]. For instance, ethylene (ET), gibberellin (GA), brassinosteroids (BRs), jasmonic acid (JA), and strigolactones (SLs) have been shown to be involved in the regulation of rice mesocotyl elongation and seedling emergence from the soil [7,8,9,10,11,12]. GA promotes cell elongation by altering the orientation of cell microtubules and enhancing pectin methylesterification [11,13,14]. It has been demonstrated that an increase in covering depth induces the production of ethylene, which activates GA synthesis-related genes (e.g., *SD1*) through transcription factors such as *OsEIL1*, thereby promoting mesocotyl elongation [15]. Auxin (IAA) predominantly regulates the activity of cell wall-loosening enzymes, thereby maintaining the cell wall in a sustained relaxed state, and promotes mesocotyl elongation through cell growth [6]. BRs promote rice mesocotyl elongation, primarily by enhancing cell division, while JA and SLs inhibit mesocotyl elongation [7,8].

Regarding genetic factors, more than 60 quantitative trait loci (QTLs) involved in regulating mesocotyl length have been identified. Only six genes, namely, *GY1*, *OsGSK2*, *OsSMAX1*, *OsPAO5*, and *SD1*, have been cloned and validated; all of them are directly or indirectly involved in plant hormone signaling pathways that regulate mesocotyl elongation. Xiong et al. (2017) cloned the mesocotyl elongation gene *GY1*, which acts on *OsEIN2*-mediated ethylene signal transduction to suppress the expression of JA biosynthesis-related genes, thereby regulating mesocotyl elongation [7]. *OsGSK2* regulates mesocotyl elongation by coordinating SL and BR signaling to promote or inhibit the phosphorylation of cyclin *CYCU2* [8]. *OsSMAX1* regulates mesocotyl elongation through the synergistic action of the karrikin and SL signaling pathways [9]. As a negative regulator of *OsSMAX1*, *OsD14L* has also been demonstrated to modulate mesocotyl elongation, with its knockout mutant exhibiting a dramatically elongated mesocotyl phenotype, further highlighting the critical role of the karrikin/SL signaling module in controlling this trait [16]. *OsPAO5* functions as a negative regulator of mesocotyl elongation. Compared with the wild type, the *OsPAO5* knockout mutant exhibits longer mesocotyls and higher ethylene production [10]. The *SD1* gene (the “Green Revolution” gene) activates GA synthesis via ethylene signaling under soil cover stress, thereby indirectly promoting mesocotyl elongation [15]. The superior haplotypes of *SD1*, *GY1*, *OsGSK2*, *OsPAO5*, and *OsSMAX1* exhibit significant additive effects in the tropical (TROP) and temperate (TEMP) *japonica* rice subgroups, which are the two widely recognized ecotype subgroups of *japonica* rice [17]. These genetic variations can be applied in molecular marker-assisted selection breeding to cultivate germplasm with long mesocotyls that can be used with dry direct-seeding technology.

In addition, the phenylpropanoid biosynthesis pathway, particularly its lignin biosynthesis branch, is a well-characterized regulator of cell wall mechanical properties via lignin deposition and plays a critical role in modulating maize mesocotyl elongation under deep-seeding stress [18,19,20]. As a conserved signal transduction module and core hub for integrating environmental and hormonal signals [21,22,23,24,25], the Mitogen-Activated Protein Kinase (MAPK) cascade modulates mesocotyl elongation through extensive crosstalk with multiple hormone pathways, as evidenced by its direct phosphorylation of key hormone signaling regulators [26,27,28]. Diterpenoid biosynthesis, relying on the plastidial 2-C-methyl-D-erythritol 4-phosphate (MEP) and cytoplasmic mevalonate (MVA) pathways that produce the universal precursor geranylgeranyl pyrophosphate (GGPP), modulates mesocotyl elongation predominantly via its core GA biosynthesis branch, with deep-sowing-induced dark conditions promoting bioactive GA synthesis by upregulating mesocotyl MEP pathway genes to boost the precursor supply [29,30,31,32]. Despite the well-documented significance of the established roles of phytohormone signaling, a limited set of cloned regulatory genes, and the functional relevance of the phenylpropanoid biosynthesis, MAPK signaling, and diterpenoid biosynthesis pathways in mesocotyl elongation across cereal crops, and the dynamic transcriptomic signatures and core regulatory genes of these three pathways that govern rice mesocotyl elongation in response to gradient sowing depths remain largely uncharacterized.

The present study demonstrated that the ML and mesocotyl cell length of rice cultivar Zhengdao 209 increased with increasing sowing depth. To determine the key pathways and genes affecting rice ML under deep-seeding conditions, the transcriptomic changes in Zhengdao 209 under three sowing depths (3, 5, and 7 cm) were analyzed using RNA sequencing (RNA-seq). According to the results of Gene Ontology (GO) and Kyoto Encyclopedia of Genes and Genomes (KEGG) enrichment analyses combined with weighted gene co-expression network analysis (WGCNA) and hierarchical clustering analysis (HCA), phenylpropanoid biosynthesis processes, the mitogen-activated protein kinase (MAPK) signaling pathway, and diterpenoid biosynthesis processes were selected for further study to identify the key regulators promoting ML. The objective of this study was to comprehend the molecular basis underlying the regulation of mesocotyl elongation by covering depth, with the aim of identifying genes associated with mesocotyl elongation and deep-sowing tolerance for genetic improvement. This study contributes to a more comprehensive understanding of the potential mechanisms that underpin deep-seeding tolerance in rice.

## 2. Materials and Methods

### 2.1. Plant Materials and Growth Conditions

The test material used in this study was Zhengdao 209, a *japonica* rice variety bred by the Institute of Food Crops, Henan Academy of Agricultural Sciences. The seeds were pre-processed as described by Liu et al. (2025) [33]. Zhengdao 209 seeds were treated at 45 °C for 48 h to break seed dormancy, then disinfected with a sodium hypochlorite solution, rinsed three times with sterile water, and soaked in water for 48 h. The rice seedlings were sown in soil contained in plastic trays with dimensions of 440 mm (length) × 330 mm (width) × 170 mm (height) in a growth chamber with 16 h light/8 h dark cycle at 28 °C at the Henan Academy of Agricultural Sciences (Zhengzhou, Henan, China). The soil was paddy soil, with a pH of 6.04, organic matter content of 21.21 g/kg, total nitrogen content of 1.27 g/kg, total phosphorus content of 0.88 g/kg, total potassium content of 18.4 g/kg, alkaline-hydrolyzable nitrogen content of 119.52 mg/kg, available phosphorus content of 55.24 mg/kg, and available potassium content of 187.08 mg/kg. Three sowing depths (3, 5, and 7 cm) were established, with the 3 cm depth serving as the control treatment. Each plastic tray was equally divided into three independent parts (330 mm × 120 mm), each of which was assigned to one of the three sowing depths. 24 viable seeds were sown in each part with a plant and row spacing of approximately 36 mm × 30 mm, and three independent plastic trays were set as biological replicates for the assay (Supplementary Figure S1).

### 2.2. Measurement of ML

To measure the ML of Zhengdao 209, the rice seeds were sown at depths of 3, 5, and 7 cm in soil contained within plastic trays. Eight days later, each rice seedling was carefully excavated and washed, and the ML was measured with a ruler. The experiment was carried out with three biological replicates. For each replicate, at least 6 rice seedlings were measured.

### 2.3. Cytological Observation of Rice Mesocotyl

Eight-day-old rice seedlings were used for measuring the length of the mesocotyl cells. For each seedling, a longitudinal section was made manually from the middle part of the mesocotyl. Then, the sections were observed and photographed with a microscope (E100; Nikon, Tokyo, Japan).

### 2.4. RNA Extraction

Five representative plants with uniform growth were collected from each of the three independent pots to generate three independent biological replicates at three sowing depths, and the total RNA for the RNA-Seq and quantitative real-time polymerase chain reaction (qRT-PCR) assays was separately isolated from the 8-day-old rice mesocotyl using the TRIzol reagent (Invitrogen Life Technologies, Carlsbad, CA, USA). Additionally, the quality of the RNA was assessed using a NanoDrop spectrophotometer (Thermo Fisher Scientific, Waltham, MA, USA).

### 2.5. Library Construction, Sequencing, and Sequence Analysis

Random oligonucleotides and SuperScript II were used to synthesize the first-strand cDNA. RNA sequencing was performed using the Illumina NovaSeq 6000 platform (Shanghai Personal Biotechnology Co. Ltd., Shanghai, China). The sequenced RNA data were then transformed by the software in the sequencing platform, and the raw data were generated. The raw data were filtered using the Cutadapt (v1.15) software to obtain clean and high-quality sequence data for further analysis.

### 2.6. Bioinformatics Analysis

The reference genome (IRGSP-1.0) and gene annotation files were downloaded from the Genome website (https://rapdb.dna.affrc.go.jp/). HISAT2 v2.0.5 was used to map the clean data to the reference genome, and HTSeq (v0.9.1) was used to compare the read count values for each gene with its original expression level. Gene expression levels were then standardized by fragments per kilobase per million reads (FPKM). The R package pheatmap (v1.0.8) was used to perform clustering analysis of the genes in the different samples.

We mapped all the genes to terms in the GO database and calculated the number of the differentially expressed genes (DEGs) for each term. In addition, we used the clusterProfiler (v3.4.4) software to conduct KEGG pathway enrichment analysis. Transcription factors and their families were predicted using PlantTFDB (v5.0) (http://planttfdb.gao-lab.org/).

### 2.7. qRT-PCR Assays

About 2 µg of total RNA was reverse transcribed into cDNA using a reverse transcriptase kit (TaKaRa, Shiga, Japan). qRT-PCR was then performed using SYBR Premix Ex Taq (TaKaRa, Shiga, Japan) on a LightCycler^®^96p (Roche, Basel, Switzerland). *OsACTIN* (*LOC_Os03g50885*) was used as an internal control. The experiments were performed with three biological replicates. The relative expression levels of all target genes under the different conditions were calculated using the 2^−ΔΔCT^ method, as described by Wang et al. (2012) [34]. All the primers used for the qRT-PCR are listed in Supplementary Table S11.

### 2.8. WGCNA Assays

Weighted gene co-expression network analysis was performed using the R package “WGCNA” (v1.72-5) to identify gene modules associated with rice mesocotyl elongation, following the standard workflow with minor optimizations. First, the cleaned transcriptome data (after removing genes with low expression (an average FPKM < 1) from all samples) were used to construct the co-expression network. A soft threshold power (β = 8) was selected based on the criterion of approximate scale-free topology (R^2^ > 0.85), transforming the pairwise gene expression correlations into weighted adjacencies. Gene modules were then identified using the dynamic tree cutting algorithm with the following parameters: minimum module size = 30, cut height = 0.25, and mergeCutHeight = 0.25. Modules exhibiting high similarity (Pearson correlation coefficient (r) > 0.75 between module eigengenes (MEs)) were then merged to eliminate redundancy. To link modules to the target trait (ML), the correlation between each ME and ML was calculated. Modules with |r| > 0.8 and < 0.01 were defined as “ML-associated key modules”. Hub genes in key modules were screened based on two criteria: gene significance (GS, the absolute correlation between gene expression and ML) greater than 0.7, and module membership (MM, the absolute correlation between gene expression and ME) greater than 0.8. The biological roles of the key modules were determined via GO and KEGG enrichment analyses using the “clusterProfiler” package (v4.6-2).

### 2.9. HCA Assays

A hierarchical clustering analysis was performed using R software (v4.3.2) and the “stats” and “pheatmap” packages (v1.0.12) to characterize the expression patterns of the DEGs associated with rice mesocotyl elongation. First, the expression values (FPKM) of the DEGs (genes with |log_2_fold change (FC)| > 1 and false discovery rate (FDR) < 0.05) were normalized using Z-score transformation (per gene) to eliminate differences in gene scale, thereby ensuring reliable clustering. HCA was then conducted based on the normalized expression matrix using two core parameters: (1) Euclidean distance, which is used to quantify the similarity between gene expression profiles, and (2) Ward.D2, which minimizes the within-cluster variance to ensure compact and distinct clusters. The clustering results were visualized as a heatmap, with the rows representing DEGs and the columns representing the samples (different sowing depths). Tree dendrograms were generated for the genes and samples to reflect the clustering relationships. A cut-off height of 0.6 on the gene dendrogram was used to delineate gene clusters, with a minimum cluster size of 30 genes, to avoid trivial groups. The functional homogeneity of each gene cluster was verified using GO/KEGG enrichment analyses with the “clusterProfiler” package (v4.6-2). Clusters with enriched terms related to cell elongation, cell wall modification, or hormone signaling were defined as “functionally relevant clusters” for subsequent analysis.

### 2.10. Statistical Analyses

The data were analyzed using IBM SPSS Statistics 21 (IBM Corp., Chicago, IL, USA, https://www.ibm.com/products/spsss-tatistics, accessed on 10 June 2025) and graphed with GraphPad Prism 7 (Systat Software, Richmond, CA, USA, https://www.graphpad.com/). Data were analyzed using independent Student’s *t*-tests at a significance level of * *p* < 0.05 and ** *p* < 0.01 vs. the control, as well as one-way ANOVA with Dunnett’s multiple comparison tests (*p* < 0.05). Values are presented as the means ± standard deviation of at least 3 biological replicates for each measurement.

## 3. Results

### 3.1. ML Varies with Different Sowing Depths

**Figure 1:**
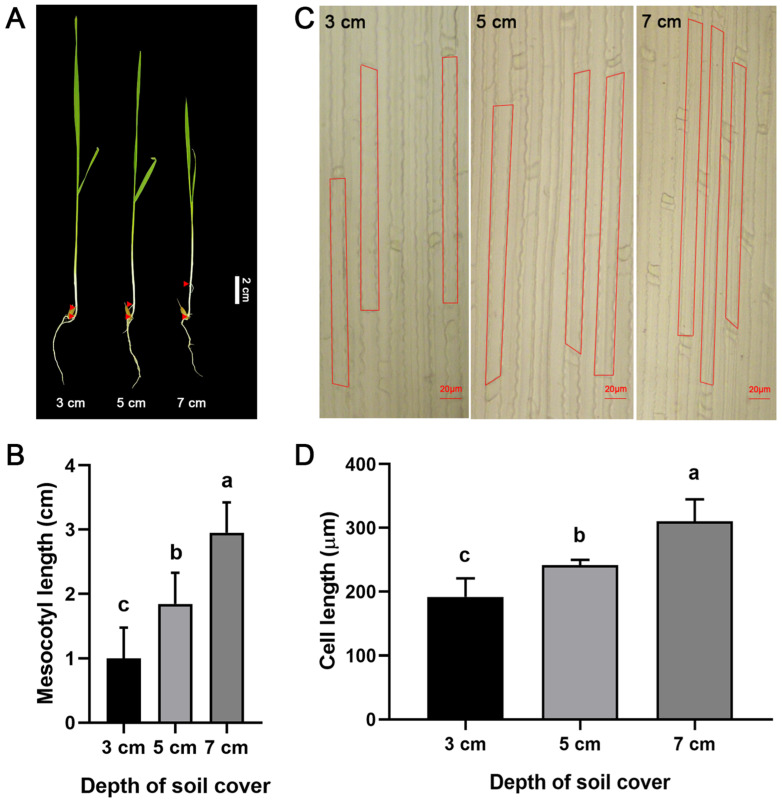
Variation in ML and mesocotyl cell length with sowing depth. (**A**) Mesocotyl morphology of the indicated plants sown at different depths. The two red triangular regions in A indicate the mesocotyl, Bar = 2 cm for (**A**). (**B**) Mesocotyl length statistics at the different sowing depths. Different letters indicate statistically significant differences between different groups, as determined by one-way ANOVA with Dunnett’s multiple comparison test (*p* < 0.05). (**C**) Mesocotyl cell lengths at the different sowing depths. The red rectangles in C characterize the single cells of the mesocotyl, Bar = 20 μm for (**C**). (**D**) Mesocotyl cell length statistics at the different sowing depths. Different letters indicate statistically significant differences between different groups according to one-way ANOVA with Dunnett’s multiple comparison test (*p* < 0.05).

After an 8-day growth period in the growth chamber, the ML of the Zhengdao 209 seedlings sown at three different depths were measured. As shown in Figure 1, at the 3 cm sowing depth, the ML was short, ranging from 0.5 to 1.5 cm; at 5 cm, it ranged from 1.3 to 2.4 cm; and at 7 cm, it exceeded 2.5 cm, reaching a maximum length of 3.4 cm. The ML increased with sowing depth from 3 cm to 7 cm (Figure 1A,B). Significant differences in mesocotyl cell length were also observed, with the greatest mesocotyl cell length observed at a sowing depth of 7 cm, followed by 5 cm, while the shortest length was observed at 3 cm (Figure 1C,D). Overall, the ML and mesocotyl cell length varied in similar manners under the different sowing depth conditions.

### 3.2. Transcriptome Sequencing and Quality Control Analysis

**Figure 2:**
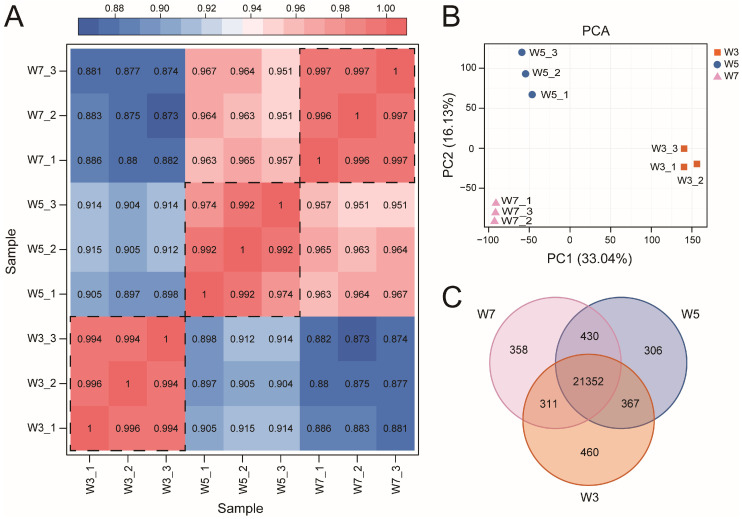
Transcriptome sequencing and quality control. (**A**) r analysis of biological replicates of rice samples across different sowing depths. The area within the dashed lines indicates the r among the three biological replicates at each sowing depth, the gradient from dark blue to dark pink indicates increasing correlation strength. (**B**) PCA of gene expression data from samples subjected to different sowing depths. (**C**) Number of expressed genes under different sowing depth conditions. W3, W5, and W7 represent sowing depths of 3, 5, and 7 cm, respectively.

To gain insights into the molecular mechanisms underlying the regulation of rice ML in response to deep sowing, we conducted transcriptome sequencing on Zhengdao 209 plants sown at depths of 3, 5, and 7 cm, with three biological replicates for each depth. A total of nine cDNA libraries (W3_1–W3_3, W5_1–W5_3, and W7_1–W7_3) were constructed for transcriptome sequencing. The raw data ranged from 41.1 to 51.5 megabase pairs (Mbp) per library. After filtering the nine samples based on quality, clean data were obtained, which ranged in size from 21.5 to 25.6 Mbp per library. These reads were aligned to the rice reference genome with an average unique alignment rate of 97.34%. Additionally, the filtered RNA-seq data for all the samples exhibited Q30 ratios ranging from 93.78% to 94.87% (Supplementary Table S1). Calculating r revealed strong correlations between biological replicates of rice samples across different sowing depths, meeting the requirements for replicate correlation (r > 0.97) (Figure 2A). Dimensionality reduction via principal component analysis (PCA) of the gene expression data revealed distinct clustering of samples from the different sowing depths (Figure 2B). These results indicated that the sequencing data were of a high enough quality to be used for further analysis. A total of 23,584 expressed genes (FPKM ≥ 1) were identified across the three sowing depth conditions (Supplementary Table S2). Of these, 21,352 genes were co-expressed across all three conditions (W3, W5, and W7), while 460, 306, and 358 genes were specifically expressed under the W3, W5, and W7 conditions, respectively (Figure 2C).

### 3.3. Identification of DEGs in Different Group Comparisons

**Figure 3:**
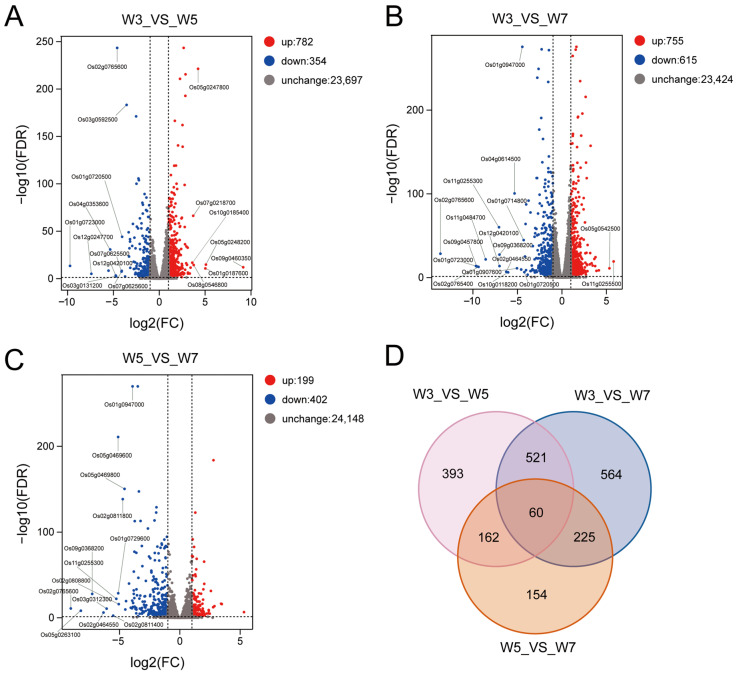
Number of DEGs in the comparisons of the three groups. (**A**–**C**) Number of up- and downregulated DEGs between 3 and 5 cm (**A**), 3 and 7 cm (**B**), and 5 and 7 cm (**C**) sowing depths. (**D**) Venn diagrams of the DEGs from the three comparisons. W3, W5, and W7 represent sowing depths of 3, 5, and 7 cm, respectively.

We performed comparisons of the transcriptome profiles at the three sowing depths (W3 vs. W5, W3 vs. W7, and W5 vs. W7). Then, DESeq (v1.30.0) was used to analyze the difference in gene expression. Based on the criteria of |log_2_FC| > 1 and FDR < 0.05, we identified 1136 DEGs between W3 and W5, of which 782 were upregulated and 354 were downregulated; 1370 DEGs were identified between W3 and W7, of which 755 were upregulated and 615 were downregulated; and 601 DEGs were identified between W5 and W7, of which 199 were upregulated and 402 were downregulated (Figure 3A–C, Supplementary Table S3). Additionally, common 60 DEGs were detected between all comparisons (Figure 3D).

**Figure 4:**
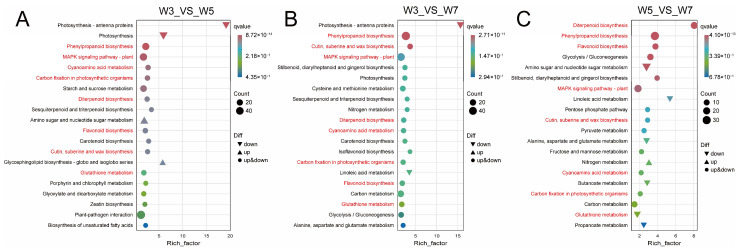
The top 20 most enriched KEGG pathway categories among the DEGs for W3 vs. W5 (**A**), W3 vs. W7 (**B**), and W5 vs. W7 (**C**). W3, W5, and W7 represent sowing depths of 3, 5, and 7 cm, respectively. Red font indicates the pathways shared by all three comparison groups, while black font indicates the pathways shared by one or two of the comparison groups.

Furthermore, to determine the functional roles of the DEGs, gene set enrichment analysis was used to identify the functional gene categories (GO terms) and the significant biological processes (Supplementary Table S4). The significantly enriched GO categories in all the comparisons were carbohydrate metabolic process, cell wall, and oxygen oxidoreductase activity (Supplementary Figure S2 and Table S5). In addition, the significantly enriched KEGG pathways were identified using gene set enrichment analysis. The key metabolic pathways in all three comparisons were phenylpropanoid biosynthesis, cutin suberine and wax biosynthesis, plant MAPK signaling pathway, diterpenoid biosynthesis, cyanoamino acid metabolism, carbon fixation in photosynthetic organisms, flavonoid biosynthesis, and glutathione metabolism (Figure 4, Supplementary Tables S6 and S7).

### 3.4. WGCNA of Transcriptome Data from Different Sowing Depth Conditions

**Figure 5:**
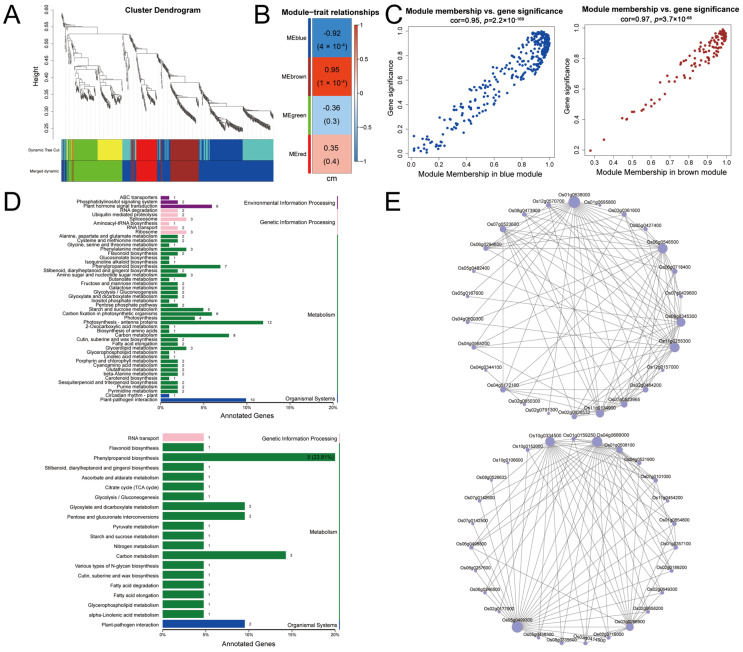
WGCNA of DEGs in the mesocotyls of rice cultivar Zhengdao 209 seedling sown at different depths. (**A**) Cluster dendrogram of DEGs. The genes were clustered based on their expression profiles, and co-expression modules (marked with distinct colors) were identified via dynamic tree cutting. The “Merged dynamic” bar represents the final integrated modules after merging highly similar ones. (**B**) Module–trait relationships between module eigengenes (MEs) and ML. Each cell shows the r (top) and the corresponding *p*-value (bottom) of the ME (of a color-labeled module) with ML. The color gradient reflects the strength and direction of the correlation. (**C**) Scatter plots of module membership (MM) vs. gene significance (GS) for the blue (left) and brown (right) modules. GS denotes the correlation between individual gene expression and ML; MM denotes the correlation between individual gene expression and the ME of the corresponding module. High linear correlations (cor > 0.95) confirm that the genes in these modules are closely associated with ML variation. (**D**) KEGG enrichment analysis of the blue (top) and brown (bottom) modules. The bars represent the proportion of annotated genes in each enriched pathway, with different colors classifying pathways into functional categories; the numbers on the bars indicate the number of annotated genes in each pathway. (**E**) Co-expression network for the blue (top) and brown (bottom) modules. Nodes represent genes, and edges represent weighted co-expression relationships; node size is positively correlated with gene connectivity (hub degree).

WGCNA was applied to investigate gene expression differences between the different sowing depths, yielding four genetic modules (Figure 5A,B). Module membership in the blue module exhibited a significant negative correlation with gene significance, whereas the brown module exhibited a significant positive correlation (Figure 5C). KEGG enrichment analysis was performed on the genes in the blue and brown modules and 48 and 20 KEGG pathways were obtained, respectively (Figure 5D). Among these, DEGs were highly enriched in the phenylpropanoid biosynthesis, carbon metabolism, photosynthesis—antenna protein, and plant–pathogen interaction pathways. Hub gene mining analysis was then performed on these two modules using the MCODE package (v2.0.3) in Cytoscape (v3.10), resulting in a network containing 26 (top) and 27 (bottom) hub genes, respectively (Figure 5E, Supplementary Table S8).

### 3.5. Spatial Functional Characteristics of Different Sowing Depths

**Figure 6:**
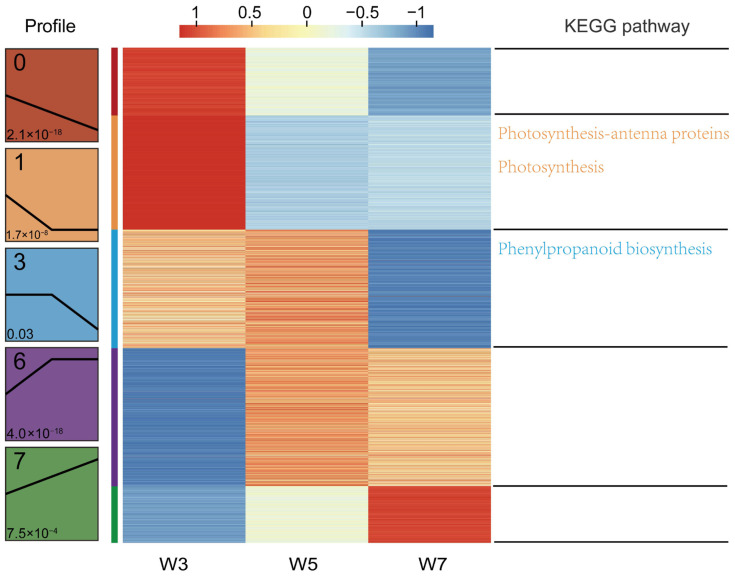
HCA-based expression profile clustering and KEGG pathway enrichment of DEGs in mesocotyls of rice plants sown at different depths. (**Left**): Co-expression patterns of the genes in the five modules. Clusters of genes associated with similar biological processes are grouped according to the degree of enrichment, as indicated on (**Right**). The red, orange, blue, purple, and green bars correspond to the genes enriched in the five modules. Each column within the sowing depth regions corresponds to a different sowing depth. The red and blue boxes indicate genes with increased and decreased abundances, respectively. W3, W5, and W7 represent sowing depths of 3, 5, and 7 cm, respectively.

To delineate the distinct features of gene expression and their biological relevance in rice mesocotyls under different sowing depths, HCA and KEGG pathway enrichment analysis were performed. Five significant expression profiles were identified across the eight sowing depth gradients (Figure 6, Supplementary Figure S3). These region-specific genes were linked to different sowing depths (Figure 1A). Profiles 0 and 1 exhibited the highest expression in W3, and decreased with deeper depths (Profile 0: *p* = 2.1 × 10^−18^; Profile 1: *p* = 1.7 × 10^−8^). These profiles were enriched with genes involved in photosynthesis antennae and photosynthesis pathways, which likely support mesocotyl elongation via the light-driven energy supply. Profile 3 peaks in W5 and declines in W7 (*p* = 0.03) and is enriched with genes involved in phenylpropanoid biosynthesis, which regulates cell wall lignin deposition and balances the mechanical support and extensibility required for moderate sowing. Profiles 6 and 7 exhibited the highest expression in W7 (Profile 6: *p* = 4.0 × 10^−18^; Profile 7: *p* = 7.5 × 10^−4^). Although they are not annotated here, their depth-dependent dynamics suggest their important roles in adapting to deep-sowing stress, complementing core mesocotyl elongation processes (Figure 6). In conclusion, photosynthesis—antenna proteins, photosynthesis pathways, and phenylpropanoid biosynthesis may be involved in mediating deep-sowing tolerance in rice.

### 3.6. Identification of the Genes Involved in Phenylpropanoid Biosynthesis Processes, the MAPK Signaling Pathway, and Diterpenoid Biosynthesis Processes

**Figure 7:**
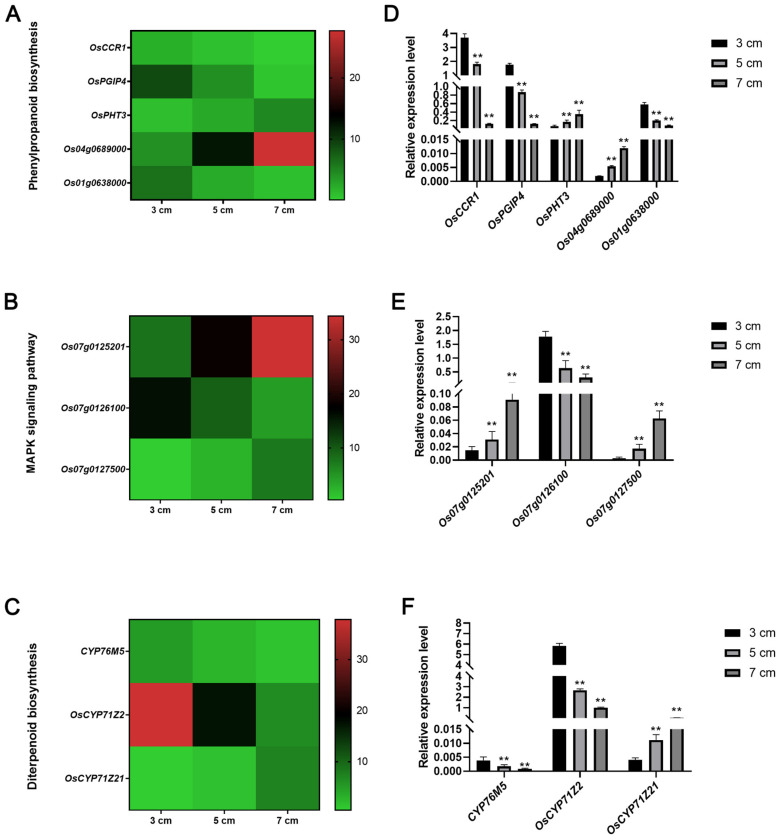
Expression pattern analysis of related genes. (**A**–**C**) Heat map showing changes in the expression of key genes in each comparison group. (**D**–**F**) qRT-PCR analyses of genes. The experiments were performed three times. The results are presented as the means ± SDs of 3 independent replicates. ** *p* < 0.01 vs. 3 cm, analyzed by Student’s *t*-test.

As phenylpropanoid biosynthesis processes, the MAPK signaling pathway, and diterpenoid biosynthesis processes are closely associated with ML under deep-sowing conditions, we analyzed the expression patterns of the related genes using transcriptome analysis. Totals of 110, 97, and 38 DEGs involved in the MAPK signaling pathway, phenylpropanoid biosynthesis, and diterpenoid biosynthesis, respectively, were detected between the different sowing depths (Supplementary Table S9). Regarding phenylpropanoid biosynthesis process genes, the heatmap showed that the expression levels of *OsPHT3* and *LOC_Os04g59260* were upregulated, while those of *OsCCR1*, *OsPGIP4*, and *LOC_Os01g45110* were downregulated with increasing sowing depth (Figure 7A). Among the genes involved in the MAPK signaling pathway, *LOC_Os07g03319* and *LOC_Os07g03580* exhibited increased expression, while *LOC_Os07g03409* was markedly downregulated with increasing sowing depth (Figure 7B). Among the genes involved in diterpenoid biosynthesis processes, significantly lower expression of *OsCYP76M5* and *OsCYP71Z2*, and higher expression of *OsCYP71Z21* were observed under deep-sowing conditions compared with the 3 cm control (Figure 7C). We then conducted qRT-PCR assays to verify the reliability of the transcriptome data. The results agreed with the RNA-seq findings (Figure 7D–F), indicating that our transcriptomic data were highly reliable. Overall, these findings imply that genes linked to phenylpropanoid biosynthesis, the MAPK signaling pathway, and diterpenoid biosynthesis might contribute to the promotion of rice mesocotyl elongation under deep-sowing conditions.

## 4. Discussion

The present study systematically investigated the molecular mechanisms underlying rice mesocotyl elongation under different sowing depth conditions (3, 5, and 7 cm) using the cultivar Zhengdao 209. Using transcriptome sequencing, WGCNA, HCA, and qRT-PCR validation, important pathways and candidate genes regulating ML under deep-sowing conditions were identified, providing novel insights into the deep-sowing tolerance of direct-seeded rice. Previous studies have demonstrated that mesocotyl elongation is mainly driven by cell elongation and moderate cell division, with cell elongation being the dominant factor [35]. Cell wall remodeling and loosening are essential physiological processes underlying mesocotyl elongation, as they facilitate cellular expansion without inflicting structural damage [28]. Consistent with earlier findings, our results corroborated that ML and mesocotyl cell length significantly increased with increasing sowing depth, with the longest ML observed at a 7 cm sowing depth (Figure 1A,B). This finding suggests that mesocotyl elongation is a crucial adaptive strategy for rice seedling emergence under deep-sowing stress [3,5,6].

HCA classified all DEGs into eight sowing depth-dependent expression patterns (Figure 6, Supplementary Figure S3 and Table S7), which were further grouped into three core regulatory modules highly correlated with the adaptive growth requirements of rice mesocotyl under different sowing depths. The first is the gradient-dependent continuous response module, containing genes continuously activated (profile 7) or repressed (profile 0) with increasing sowing depth. The continuously upregulated genes are likely core positive regulators mediating deep-sowing-induced mesocotyl elongation, whose sustained transcriptional activation provides molecular driving force for soil penetration. In contrast, the continuously downregulated genes are mainly involved in basal mesocotyl growth under shallow sowing, and their specific repression under deep-sowing stress facilitates the growth pattern transition for deep-sowing tolerance. The second is the transition-stage balance regulatory module, including four gene sets with expression peaking/valleying, or being specifically activated (profile 5 and profile 6)/repressed (profile 1 and profile 2) at 5 cm sowing depth. These genes reflect the transcriptional balance of mesocotyl during the shallow-to-deep sowing transition, and may act as core signal transduction nodes to perceive environmental cues (e.g., mechanical pressure, light gradient), serving as key hubs for mesocotyl growth state switching across sowing depths. The third is the deep-sowing-specific response module, comprising two gene sets exclusively activated (profile 4) or repressed (profile 3) under 7 cm deep sowing, with no expression changes under shallow/moderate conditions. These genes form a deep-sowing-specific transcriptional regulatory unit, and their strict expression specificity suggests they are key mediators of mesocotyl deep-sowing adaptation and core candidates for subsequent functional validation.

Notably, a large number of transcription factors (TFs) were identified among the DEGs (Supplementary Table S10), including multiple well-documented TF members (e.g., *OsNAC29* [29], *OsWRKY24* [36], *OsbHLH035* [37], *OsARF17* [38]) that have been functionally validated to regulate rice mesocotyl elongation via mediating phytohormone signal crosstalk and cell wall remodeling, as well as a set of novel TFs with unreported functions (e.g., *ARF*, *bZIP*, *C2H2*, *MYB*, *NAC*), which represent promising candidate regulators for deciphering new transcriptional regulatory mechanisms of deep-sowing tolerance in direct-seeded rice. The differential expression characteristics of the previously characterized TFs not only verified the reliability of our transcriptome data, but also expanded the functional scenario of these classical regulators, confirming that they can act as regulatory nodes to integrate environmental and hormone signals related to sowing depth and regulate mesocotyl elongation. Meanwhile, the identification of novel TFs provides brand-new candidate targets for dissecting new regulatory pathways of deep-sowing adaptation in rice mesocotyl.

Transcriptome analysis identified 60 common DEGs across all three comparisons, and functional enrichment revealed that these DEGs were primarily involved in phenylpropanoid biosynthesis, MAPK signaling, and diterpenoid biosynthesis (Figure 4 and Figure 5). The findings indicated that rice mesocotyl elongation under deep-sowing conditions is a complex process coordinated by multiple metabolic and signaling pathways. The phenylpropanoid biosynthesis pathway is well-documented to regulate cell wall mechanical properties via lignin deposition [18,19,20]. Among the diverse metabolites identified, the lignin biosynthesis branch shows the closest link to mesocotyl elongation [19,20]. In maize, the spatiotemporal regulation of lignin deposition through this pathway modulates mesocotyl cell wall mechanical properties; specifically, reduced lignin biosynthesis and accumulation reduces cell wall rigidity, thereby facilitating marked mesocotyl elongation under deep-seeding stress [18]. Consistent with this regulatory framework, the present study revealed distinct expression patterns of genes relevant to lignin metabolism and mesocotyl elongation in rice under varying sowing depths. Specifically, *OsCCR1*, a key gene in lignin synthesis, was downregulated with increasing sowing depth, indicating that the reduced lignin deposition in the mesocotyl preserves cell wall extensibility to promote mesocotyl cell and mesocotyl elongation [39,40]. In contrast, the putrescine hydroxycinnamoyl acyltransferase *OsPHT3* and *LOC_Os04g59260* were upregulated under the same conditions [41], while *OsPGIP4* (mainly expressed in callus and seeds and is a positive regulator of bacterial leaf streak) and *LOC_Os01g45110* were downregulated with increasing sowing depth [42] (Figure 7A,D). Taken together, *OsPHT3* and *LOC_Os04g59260* may positively regulate mesocotyl elongation by modulating lignin synthesis under deep-sowing conditions, whereas *OsPGIP4* and *LOC_Os01g45110* exert the opposite effect.

The MAPK signaling pathway serves as a core hub integrating environmental and hormone signals [21,22,23,24,25]. As a conserved signal transduction module, the MAPK cascade orchestrates mesocotyl elongation by engaging in extensive cross-talk with multiple hormone pathways, a mechanism supported by the observation that MAPK components directly phosphorylate key hormone signaling regulators, such as auxin influx carriers (*OsAux*/*LAX1*) [27], DELLA proteins (GA signaling repressors) [26], and *BZR1* (BR signaling transcription factor) [43]. It is noteworthy that the identification of three novel MAPK-related DEGs (*LOC_Os07g03319*, *LOC_Os07g03580*, and *LOC_Os07g03409*) with divergent expression patterns under deep-sowing conditions extends this regulatory framework. These novel MAPK-related genes may act as additional regulatory nodes within the *OsMAPKKK10–OsMAPKK4*–*OsMAPK6* module, regulating downstream cell elongation-related transcription factors or associated proteins [28]. The identification of novel MAPK-related regulatory genes enriches the current understanding of the molecular mechanisms underlying rice deep-sowing adaptation and provides several promising candidate genes for the genetic improvement of direct-seeded rice varieties to enhance seedling emergence under deep-sowing conditions. Subsequent studies will focus on testing the protein–protein interactions between these novel genes, core MAPK cascade components, and key hormone signaling regulators (e.g., *OsEIL1*, *DELLA*, and *OsAux/LAX1*), and validating their potential dual roles in MAPK-mediated transcriptional regulation and cell wall remodeling to verify our proposed regulatory mechanism and unravel the precise molecular circuitry underlying rice deep-sowing adaptation.

Diterpenoid biosynthesis relies on the plastidial MEP and cytoplasmic MVA pathways, which function to synthesize GGPP. GGPP is then converted into structurally diverse diterpenoids via diterpene cyclases/synthases. The gibberellin biosynthesis branch serves as the core regulator of mesocotyl elongation [29]. Notably, deep-sowing-induced dark conditions significantly upregulate the key MEP genes in the mesocotyl, thereby boosting GGPP production to provide the metabolic precursor for the GA precursor ent-kaurene, which is subsequently catalyzed by ent-kaurene oxidase and ent-kaurenoic acid oxidase to form bioactive GAs [30,31,32]. In the present study, several *cytochrome P450 monooxygenase* (*CYP*) genes were identified as key regulators of this process: *OsCYP71Z21* was upregulated, while *OsCYP76M5* and *OsCYP71Z2* were downregulated with increasing sowing depth (Figure 7C,F). Consistent with previous reports that *CYPs* are involved in GA precursor modification [6,35,44], these results suggest that deep-sowing-induced dark signals may reshape the diterpenoid metabolic profile by modulating the expression of these *CYP* genes, thereby fine-tuning GA biosynthesis to facilitate mesocotyl elongation under deep-sowing stress.

Studies have shown that deep sowing reduces the formation and accumulation of total lignin, resulting in decreased cell wall rigidity, thereby significantly promoting mesocotyl elongation, while photosynthesis and carbon metabolism provide the energy and carbon skeleton for mesoblastic cell elongation, promoting rapid emergence under shallow sowing conditions [20,35,43,45,46]. WGCNA identified blue and brown modules that were strongly correlated with ML and enriched in genes involved in phenylpropanoid biosynthesis, carbon metabolism, and photosynthesis antennae (Figure 5C,D). The carbon metabolism and photosynthesis pathways (enriched in Profiles 0 and 1) may supply the energy and carbon skeletons for mesocotyl elongation under shallow sowing. Meanwhile, the phenylpropanoid biosynthesis pathway (Profile 3) strikes a balance between mechanical support and extensibility under moderate sowing. Profiles 6 and 7, which were highly expressed at a 7 cm sowing depth, may represent novel deep-sowing stress-responsive genes that warrant further investigation.

This study systematically analyzed the effects of three sowing depths on mesocotyl elongation through transcriptome sequencing, identified DEGs, verified candidate genes, defined the sowing depth-dependent expression patterns of a large number of uncharacterized genes, and constructed a comprehensive transcriptome dynamic map of rice mesocotyl in response to sowing depth gradients. However, this study provides a correlative transcriptomic landscape of rice mesocotyl in response to sowing depth gradients, and cannot confirm the causal relationship between the identified DEGs/pathways and mesocotyl length variation, which is the core direction of our follow-up functional studies. There are several limitations: (1) only one rice cultivar (Zhengdao 209) was used, and thus, the results need to be verified in other genetic backgrounds; (2) functional validation (e.g., CRISPR/Cas9-mediated gene editing or overexpression assays) is necessary to confirm the roles of the candidate genes and (3) the interaction between key pathways (e.g., MAPK signaling and GA biosynthesis) remains unclear. Future research should focus on the following aspects: (1) verifying the functions of the candidate genes (e.g., *OsCCR1*, *LOC_Os07g03319*, and *OsCYP71Z21*, especially TFs that have not undergone functional validation) via genetic transformation, (2) exploring the interaction between key genes and hormones (GA, ABA, and auxin) under deep sowing conditions, and (3) targeting the identified genes in molecular breeding to develop deep-sowing-tolerant rice varieties. These efforts will accelerate the large-scale adoption of direct-seeding technology, thereby contributing to water conservation and the development of mechanized agriculture.

## 5. Conclusions

Taken together, our transcriptome data provide a comprehensive gene expression profile for Zhengdao 209 at three sowing depths that showed varying ML phenotypes, and identified candidate genes that may regulate ML. Further studies are needed to functionally validate these candidate genes and investigate their roles in regulating ML. Ultimately, these genes could be targeted in breeding programs to develop rice cultivars with longer MLs in the future.

## Data Availability

All data have been carefully presented in the main text or provided as Supplementary Materials, with the transcriptome dataset deposited in the GEO database (accession number: GSE324982). Should additional data be required, please contact the corresponding author.

## Supplementary Materials

The following supporting information can be downloaded at: https://www.mdpi.com/article/10.3390/genes17040382/s1, Figure S1: Seedling emergence and growth performance of rice cultivar Zhengdao 209 with sowing depth; Figure S2: The significantly enriched GO term for the DEGs in each comparison group; Figure S3: Hierarchical Clustering Analysis (HCA) of Expression Patterns for Differentially Expressed Genes (DEGs) in Rice Mesocotyl; Table S1: Quality and map of transcriptome sequencing data; Table S2: Raw counts of nine samples; Table S3: Differential expression genes of three comparative groups; Table S4: Annotated information for all genes; Table S5: GO enrichment analysis of differentially expressed genes in three comparative groups; Table S6: KEGG enrichment analysis of differentially expressed genes in three comparative groups; Table S7: KEGG analysis of genes in each profile of hierarchical clustering analysis (HCA); Table S8: Annotated information for hub genes; Table S9: MAPK signaling pathway, phenylpropanoid biosynthesis, and diterpenoid biosynthesis-related DEGs; Table S10: Annotated information for transcription factor; Table S11: Primers of qRT-PCR.

## Abbreviations

The following abbreviations are used in this manuscript:

BRs	brassinosteroids
DEGs	differentially expressed genes
ET	ethylene
FPKM	fragments per kilobases per million reads
GA	gibberellin
GO	Gene Ontology
HCA	hierarchical clustering analysis
IAA	auxin
JA	jasmonic acid
KEGG	Kyoto Encyclopedia of Genes and Genomes
MAPK	mitogen-activated protein kinase
Mbp	megabase pairs
ML	mesocotyl length
PCA	principal component analysis
PlantTFDB	Plant Transcription Factor Database
QTL	quantitative trait loci
qRT-PCR	quantitative real-time polymerase chain reaction
SLs	strigolactones
WGCNA	weighted gene co-expression network analysis
TROP	Tropical
TEMP	Temperate
RNA-seq	RNA sequencing
FC	fold change
CYP	cytochrome P450 monooxygenase
MEP	2-C-methyl-D-erythritol 4-phosphate
MVA	mevalonate
GGPP	geranylgeranyl pyrophosphate
Aux	auxin
TFs	transcription factors
