# Supplementary material for: Transcriptional Response of Rice Mesocotyl Elongation to Sowing Depth and Identification of Key Regulatory Factors
Source: Genes (Basel). 2026 Mar 27;17(4):382. doi: 10.3390/genes17040382 (PMC13116642; doi:10.3390/genes17040382)
Supplement: Supplementary file 1 [file genes-17-00382-s001.zip › Supplementary figures.pdf]

genes

MDPI

Article

Transcriptional Response of Rice Mesocotyl Elongation to Sowing Depth and Identification of Key Regulatory Factors

Ya Wang [1,†], Dong Liu [1,†], Mengjuan Ma [1], Ming Li [1], Jing Fu [1], Fengjiang Yu [2], Qiulin Li [2], Yuetao Wang [1], Fuhua Wang [1], Liyu Huang [2] and Haiqing Yin [1,*]

[1] Cereal Crops Research Institute, Henan Academy of Agricultural Sciences, Zhengzhou 450002, China; wangya840212@163.com (Y.W.); liudongzh@hnagri.org.cn (D.L.)
[2] School of Agriculture, Yunnan University, Kunming 650091, China
* Correspondence: wangya840212@hnagri.org.cn
† These authors contributed equally to this work.

Supplementary Figures

Figure S1. Seedling emergence and growth performance of rice cultivar Zhengdao 209 with sowing depth.

Figure S2. The significantly enriched GO term for the DEGs in each comparison group.

Figure S3. Hierarchical clustering analysis (HCA) of expression patterns for differentially expressed genes (DEGs) in rice mesocotyl under three sowing depths.

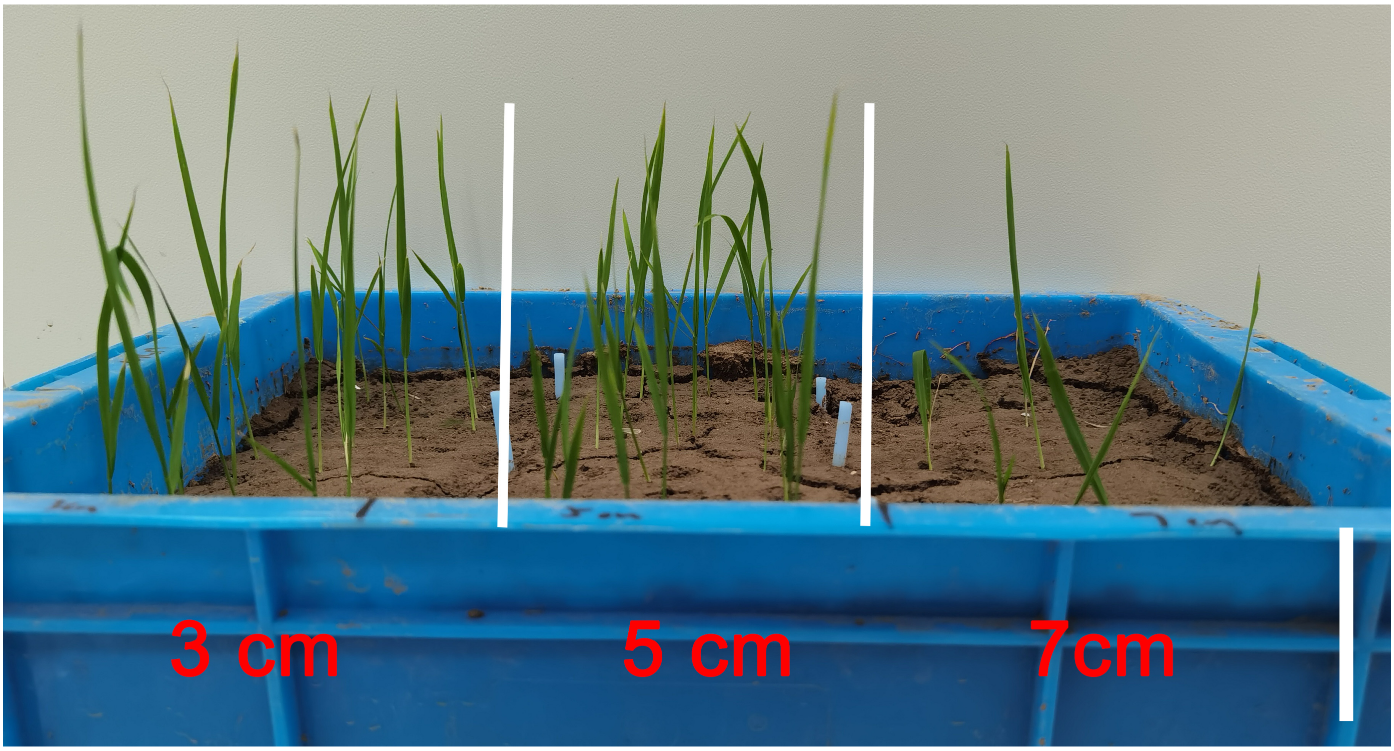

Figure S1. Seedling emergence and growth performance of rice cultivar Zhengdao 209 with sowing depth. Bar = 5 cm.

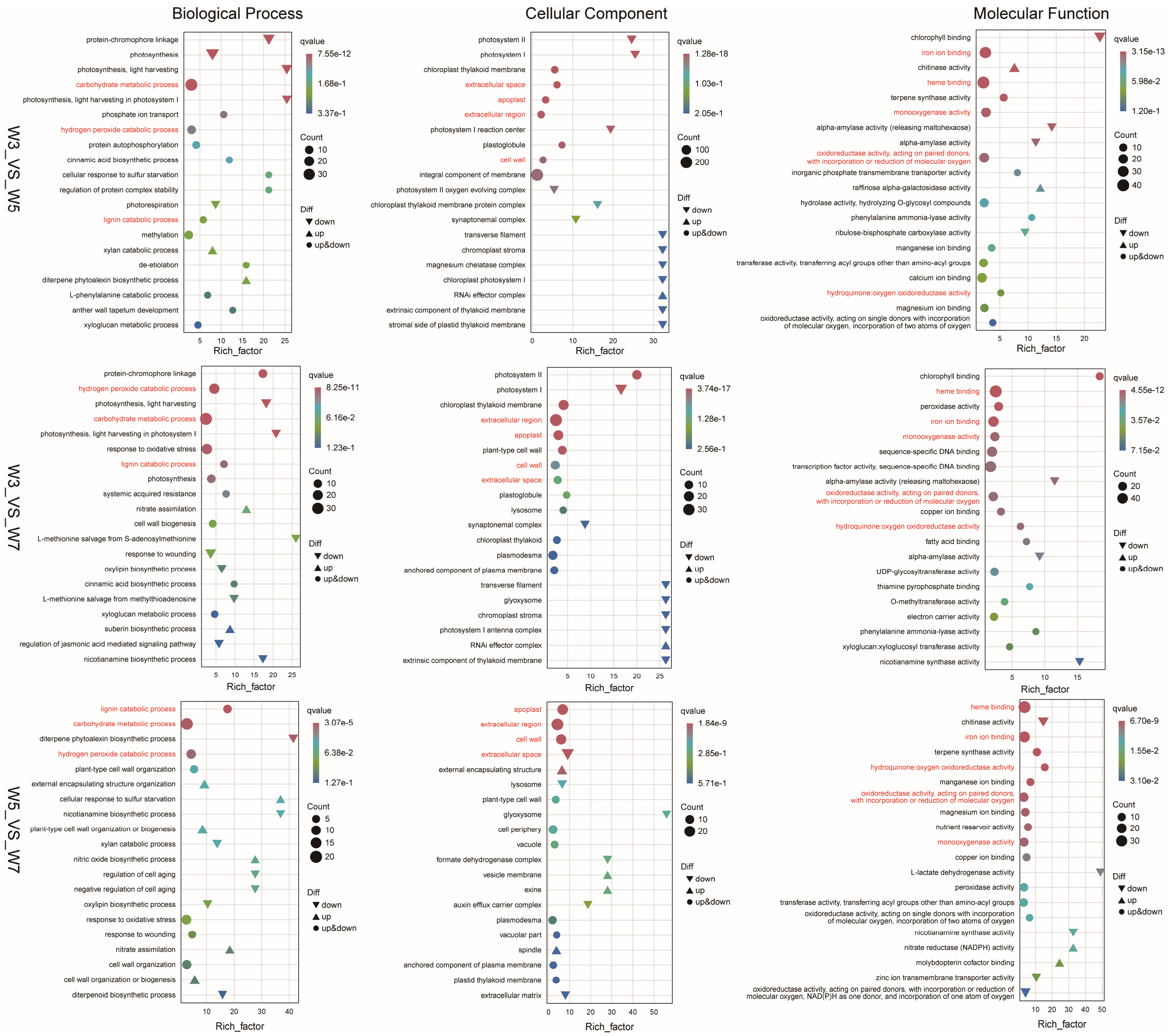

Figure S2. The significantly enriched GO term for the DEGs in each comparison group. The top 20 most enriched GO biological process categories among the DEGs for W3 vs. W5 (A), W3 vs. W7 (B), and W5 vs. W7 (C). W3, W5, and W7 represent sowing depths of 3, 5, and 7 cm, respectively.

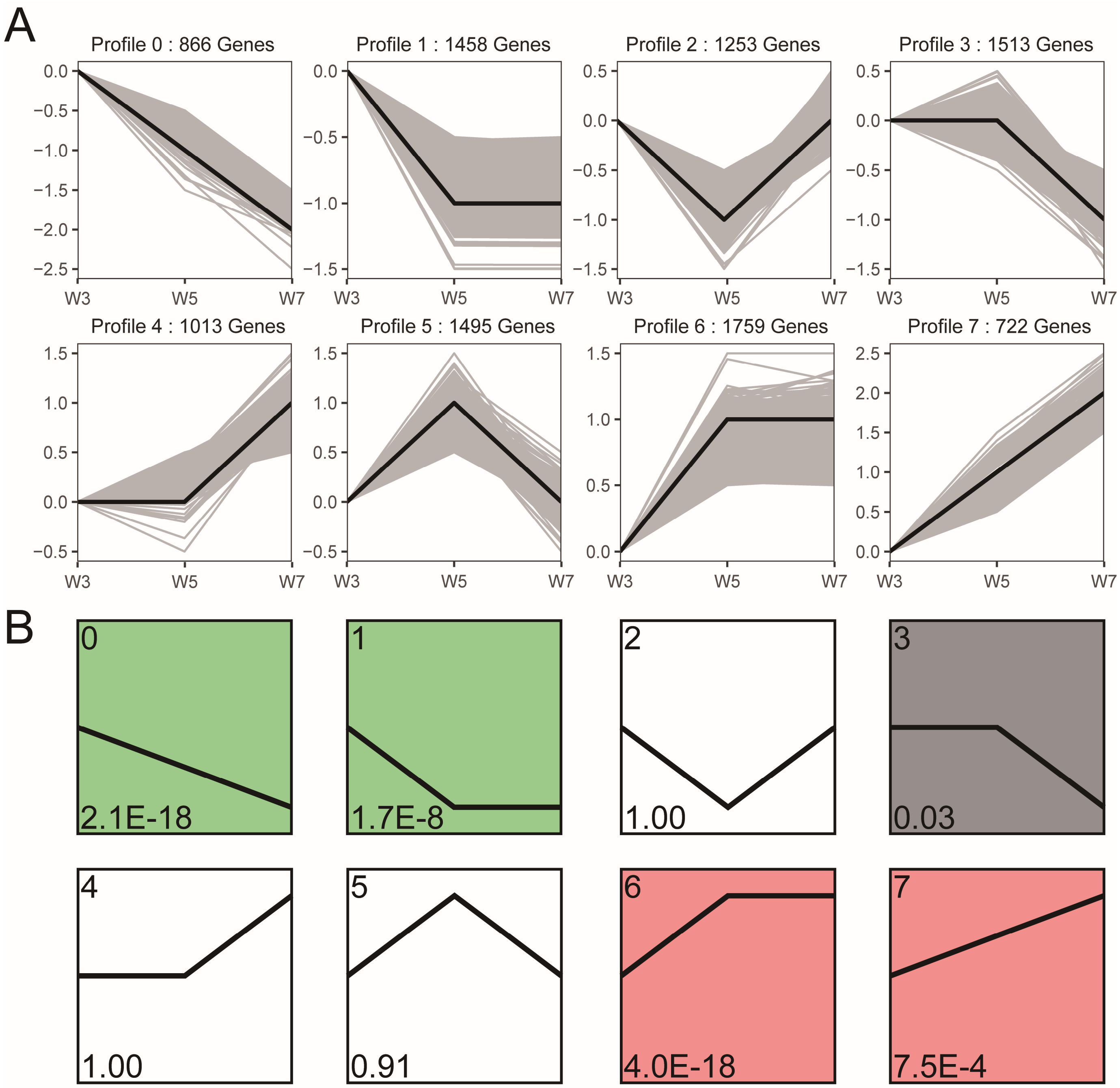

Figure S3. Hierarchical Clustering Analysis (HCA) of Expression Patterns for Differentially Expressed Genes (DEGs) in Rice Mesocotyl. (A) Detailed expression trend plots of 8 gene expression profiles (Profile 0–7). Each subplot indicates the number of genes included in the corresponding Profile. The horizontal axis represents sowing depths (W3: 3 cm, W5: 5 cm, W7: 7 cm), and the vertical axis represents normalized expression levels of genes. The gray shaded area denotes the expression fluctuation range of all genes within the same Profile. (B) Simplified schematic diagrams of each Profile. Background colors distinguish Profiles with different clustering significance, and the value in each box represents the P-value of the corresponding expression pattern (a smaller P-value indicates higher clustering significance of the gene expression pattern).
